# Anabolic androgenic steroids and cardiomyopathy: an update

**DOI:** 10.3389/fcvm.2023.1214374

**Published:** 2023-07-26

**Authors:** Kahtan Fadah, Gokul Gopi, Ajay Lingireddy, Vanessa Blumer, Tracy Dewald, Robert J. Mentz

**Affiliations:** ^1^ Division of Cardiovascular Medicine, Department of Internal Medicine, Texas Tech University Health Sciences Center El Paso, El Paso, TX, United States; ^2^ Department of Internal Medicine, The Brooklyn Hospital Center, Brooklyn, NY, United States; ^3^ Department of Cardiovascular, Heart and Vascular Institute, Kaufman Center For Heart Failure, OH, United States; ^4^ Department of Cardiovascular, Duke University Medical Center and Duke Clinical Research Institute, Durham, NC, United States

**Keywords:** anabolic androgenic steroids, cardiomyopathy, heart failure, myocardial injury, ventricular dysfunction

## Abstract

Anabolic androgenic steroids (AAS) include endogenously produced androgens like testosterone and their synthetic derivatives. Their influence on multiple metabolic pathways across organ systems results in an extensive side effect profile. From creating an atherogenic and prothrombotic milieu to direct myocardial injury, the effects of AAS on the heart may culminate with patients requiring thorough cardiac evaluation and multi-disciplinary medical management related to cardiomyopathy and heart failure (HF). Supraphysiological doses of AAS have been shown to induce cardiomyopathy via biventricular dysfunction. Advancement in imaging including cardiac magnetic resonance imaging (MRI) and additional diagnostic testing have facilitated the identification of AAS-induced left ventricular dysfunction, but data regarding the impact on right ventricular function remains limited. Emerging studies showed conflicting data regarding the reversibility of AAS-induced cardiomyopathy. There is an unmet need for a systematic long-term outcomes study to empirically evaluate the clinical course of cardiomyopathy and to assess potential targeted therapy as appropriate. In this review, we provide an overview of the epidemiology, pathophysiology and management considerations related to AAS and cardiomyopathy.

## 1. Introduction

Anabolic androgenic steroids (AAS) are a large group of molecules that involve endogenously produced androgens like testosterone and their synthetic derivatives. AAS have been approved by the Food and Drug Administration (FDA), mainly limited to treating anemia secondary to bone marrow and renal failure, hypogonadism, cancer, endometriosis, and wasting syndrome in human immunodeficiency virus infection (HIV) (1–3). Despite the widely recognized benefits of testosterone therapy in terms of enhancing sexual function, increasing muscle mass and bone density, and improving physical function in aging individuals, recent literature has brought attention to risks associated with this therapy, irrespective of its indication for use (4–6). Several studies have reported net positive effects, while others suggest potential adverse outcomes (7). Specifically, there have been contradictory findings regarding the impact of testosterone treatment on cardiovascular events, including coronary artery noncalcified plaque progression and heart failure (8, 9).

Contrary to the low frequency of usage in routine clinical practice, professional athletes and young adults between the age of 20 and 40 who train in weight lifting, bodybuilding, or martial arts, more commonly use AAS to enhance muscle mass or physical performance. While their misuse was initially limited mostly to professional bodybuilders, misuse has increased over time among recreational athletes despite being forbidden by the World Anti-Doping Agency (10).

Although most adverse events associated with AAS are non-fatal, evidence shows that chronic use of supraphysiologic doses of AAS (usually due to misuse) is linked to severe medical consequences particularly advanced heart failure (HF) (11). HF cases secondary to AAS abuse in the absence of atherosclerosis pathologies are being reported more often in recent years (12). While there are several mechanisms of action attributed to AAS induced cardiomyopathy, left ventricular (LV) dysfunction remains one of the most common abnormalities and a major risk for developing serious events.

The Testosterone Replacement therapy for Assessment of long-term Vascular Events and efficacy ResponSE in hypogonadal men (TRAVERSE) study (13) which was recently published showed that testosterone-replacement therapy in men with hypogonadism and preexisting or a high risk of cardiovascular disease was noninferior to placebo with respect to the incidence of major adverse cardiac events. However, it is important to note that adverse events resulting from misuse in patients without hypogonadism remain unknown and were not directly addressed by this trial. This article reviews the contemporary knowledge and recent understanding of AAS abuse, mechanisms leading to AAS induced cardiomyopathy and current approach to medical management.This article reviews the contemporary knowledge and recent understanding of AAS abuse, mechanisms leading to AAS induced cardiomyopathy and current approach to medical management.

### 1.1. Physiology of testosterone

Testosterone, the main male sex hormone, plays a crucial role in the development of male reproductive organs and secondary sex characteristics. It is primarily produced in the testes and predominantly circulates in the blood bound to proteins and sex hormone-binding globulin. Only a small fraction, around 1%–2%, exists in a free form (14). Once testosterone binds to androgen receptors, it initiates gene transcription that influences different tissues downstream (15).

AAS encompass a diverse range of natural and synthetic androgens that are derived from testosterone. The term “anabolic” describes their ability to promote the synthesis of complex molecules from simpler ones, facilitating tissue-building in the body. On the other hand, “androgenic” refers to their masculinizing effects. Many AAS have been created by modifying the structure of testosterone derivatives to reduce androgenic effects while maximizing the anabolic advantages (16). The anabolic effects of various AAS have many medical applications including the FDA-approved uses of oxymetholone in the treatment of congenital and acquired aplastic anemia (17), danazol in hormone-receptive endometriosis (18, 19), and testosterone undecanoate in the treatment of primary hypogonadism (20). The same anabolic properties of AAS use in medical therapy also make them particularly attractive to athletes who seek to increase muscle mass for increased strength and speed, and in individuals who desire cosmetic muscular changes (21). Table 1 shows some of the commonly used AAS and their route of intake.

**Table 1:** Commonly used AAS (15).

Oral Agents	Injectable Agents
Methandrostenolone	Testosterone esters: blend, cypionate, enanthate, heptylate, propionate
Methyltestosterone	Nandrolonr esters: decanoate, phenpropionate
Oxandrolone	Boldenone
Oxymetholone	Trebolone
Mestanolone	Stanozol
Stanazol	Dromostanolone
Ethylestrenol	Androstanolone
Fluoxymesterone	Methandrostenolone
Danazol	
Androstanolone	
Metenolone enanthate	
Mesterolone	
Chlorodehydromethyltestosterone	
Trienolone	
Enobosarm	

### 1.2. Prevalance of AAS

AAS abuse in the United States has been steadily increasing in the past 40 years (22). Although competitive athletes were among the first groups of people described to be abusing AAS for their purported performance-enhancing effects, AAS abuse spread to the general population at large by the 1970 s (23). Interest in AAS increased in the 1990 s due to improved awareness of the prevalence and adverse effects of AAS in the population, culminating in the Anabolic Steroids Control Act in 1990 which added anabolic steroids to the list of scheduled III substances (11). A 2013 study that pooled data from 10 previous studies estimated a total of 2.9–4.0 million individuals used AAS between the ages of 13–50 years (24), putting the overall lifetime prevalence at over 1% (25).

### 1.3. AAS and cardiovascular system

#### 1.3.1. Gateway to myocardial injury

Approximately 3% of AAS users have been estimated to develop acute myocardial infarction at a young age (26). AAS' role in the pathophysiology of myocardial injury is thought to be multifactorial in that it contributes to coronary vessel injury via atherosclerosis, thrombosis, and vasospasm (27). Atherosclerosis in AAS users has been directly associated with dyslipidemia via elevated low-density lipoprotein (LDL) and lowered high-density lipoprotein (HDL), lipoprotein (a) concentrations, and apolipoprotein A (ApoA levels) (28). The effects of AAS on serum lipids is dose dependent and these changes in lipids usually result in decreased regression of atherosclerotic plaques (28–31). Baggish et al. found that AAS users are more likely to develop higher coronary plaque volume compared to non-users (26). There is also a positive correlation between the duration of AAS exposure and plaque volume progression (26).

Even though there is evidence that physiologic levels of testosterone may cause arterial vasodilation (32), increased doses have been found to instead facilitate vasoconstriction (33). Further factors contributing to possible vasospasm with AAS use include the increase in norepinephrine, angiotensin II and thromboxane, all of which promote vasoconstriction (35).

AAS increases the risk of thrombosis by affecting platelets, coagulation/fibrinolysis cascade, and vascular tone. They modify prostaglandins, leading to increased platelet aggregation at sites of endothelial injury (35). Androgens stimulate the synthesis of procoagulant factors and also promote hematopoiesis, contributing to a higher thrombosis risk (31–34, 36). They alter arterial tissue structure by decreasing elastin and increasing collagen and other fibrous proteins (37, 38). AAS also enhance vascular reactivity (39) and increases vascular tone by inhibiting nitric oxide synthesis (40). This prothrombotic state, combined with age-related atherosclerosis, results in irreversible myocardial damage. The aggregates of these factors may predispose AAS users to early presentation with ST-segment elevation myocardial infarction (STEMI), or Non-ST segment elevation (NSTEMI) with or without cardiogenic shock (26, 41, 42). Table 2 demonstrates different pathways of AAS impact on the cardiovascular system.

**Table 2:** AAS impact in the cardiovascular system.

AAS effect	Pathophysiology	Outcome	Reference number
Cardiovascular system			
Atherosclerosis	Increased LDL, reduced HDL, Lpa, and ApoA, and Inflammation	Myocardial Injury	(42, 45, 48, 93)
Thrombosis	Alteration of prostaglandins, increased platelet aggregation, stimulates synthesis of pro-coagulant factors		(49, 50)
Vasospasm	Upregulation of voltage-gated calcium channels Increase in Norepinephrine, angiotension II, thromboxane		(57, 58)
Left ventricular hypertrophy	Direct stimulation of androgen receptors on cardiac myocytes Activation of RAAS Increased fibrosis ERK1/2 and mTOR activation system	Hypertrophy	(64, 65, 94)
Right ventricular Strain	Fibrosis		(67)

**Figure 1:**
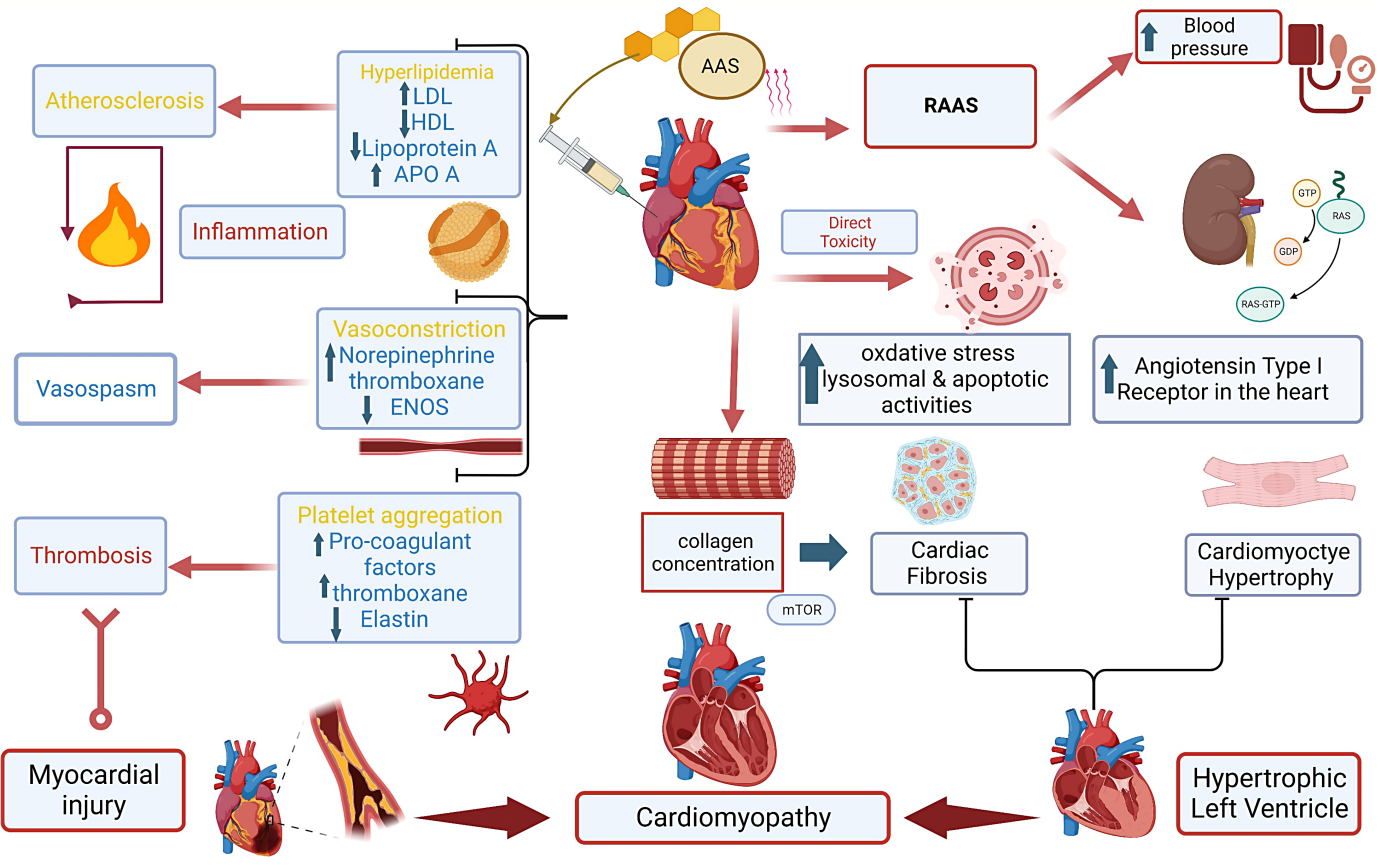
Central illustration. Anabolic androgenic steroids and mechanisms to cardiac injury leading to cardiomyopathy. Anabolic androgenic steroids (AAS), Low-density lipoprotein (LDL), High-density lipoprotein (HDL), Apolipoprotein A (ApoA), endothelial nitric oxide synthesis (ENOS).

In situations involving STEMI or NSTEMI in AAS users, additional considerations are necessary, including addressing AAS withdrawal symptoms and adapting medication to accommodate AAS-related cardiovascular issues. Individualized treatment plans and care become essential. The primary diagnostic and treatment approach for STEMI in AAS users remains coronary angiography, regardless of age. While NSTEMI in AAS patients has limited research, management is similar to individuals without AAS history and treatment decisions are based on patient-specific risk factors and presentation characteristics (43). According to the European Society of Cardiology (ESC), an immediate invasive strategy, such as percutaneous coronary intervention (PCI), should be considered within 2 h for patients with severe left ventricular dysfunction, cardiogenic shock, hemodynamic instability, life-threatening arrhythmias or mechanical complications (44). Early invasive strategies within 24 h have been shown to improve survival benefits in higher-risk patients with a Global Registry of Acute Coronary Events (GRACE) score greater than 141 (43). An invasive strategy should be considered within 72 h for patients with intermediate-risk criteria such as diabetes mellitus, renal insufficiency, prior myocardial intervention, HF with mild reduced ejection fraction, and GRACE score between 140 and 109 (43, 44). However, a conservative strategy with optimal medical therapy without PCI is acceptable in non-urgent cases, when there is no obvious coronary stenosis or when procedural risk outweighs benefits (45, 46). Figure 1. Shows AAS potential mechanism leading to cardiac injury and HF.

#### 1.3.2. Exploring the link between AAS use and the development of HF

##### 1.3.2.1. Role of AAS in left ventricular hypertrophy

The pathogenesis of HF in individuals with AAS involves biventricular dysfunction resulting from various factors that compromise mechanical and electrophysiological functioning. AAS use has been found to be associated with myocardial hypertrophy, with echocardiography results of the HAARLEM study demonstrating a positive relationship between the increase in left ventricular thickness and the supraphysiological doses of AAS administered (47). Chronic AAS use can lead to myocardial hypertrophy through various pathways. One straightforward pathway involves androgen receptors on cardiac myocytes (48). Additionally, the literature describes complex mechanisms of AAS-induced myocardial hypertrophy related to disruptions in the Renin-Angiotensin-Aldosterone System (RAAS). The RAAS can cause left ventricular hypertrophy and cardiac fibrosis through three mechanisms: elevated blood pressure, the direct action of angiotensin II on cardiac myocytes via the angiotensin type 1 (AT-1) receptor, and aldosterone-mediated effects. AAS affects all three mechanisms, promoting RAAS-mediated cardiac remodeling (49). Maintenance of systemic blood pressure is tightly balanced between the baroreceptor reflex and the Bezold–Jarisch reflex (50). AAS administration in male Wistar rats has been found to alter inflammatory cytokines and angiotensin-converting enzyme (ACE) activity, disrupting the Bezold–Jarisch reflex and leading to hypertension and subsequent cardiac hypertrophy ases ACE levels, resulting in elevated angiotensin II and activation of the AT-1 receptor, which induces myocardial hypertrophy and fibrosis independent of blood pressure (51). Chronic AAS use further upregulates the AT-1 receptor, contributing to the hypertrophic effect (52). Increased aldosterone levels stimulate oxidative stress, inflammation, and fibrosis, promoting cardiac remodeling through interactions between the mineralocorticoid receptor and AT-1 (53).

Despite the increase in myocardial thickness due to AAS use, this mechanism is not efficiently translated into increased contractility or improved performance. Much of the newly increased mass is a result of augmented matrix collagen deposition and increased fibrosis, rather than a true absolute increase in the size or number of cardiac myocytes. Molecular studies on AAS-induced hypertrophy have demonstrated an increase in the activity of the Ras-dependent extracellular signal-regulated kinase 1 (ERK1/2) and mammalian target of rapamycin (mTOR) activation system, leading to increased fibrosis but no changes in alpha myosin heavy chain (α-MHC) expression, which is responsible for contractility (54). These changes are also associated with remodeling of gap junctions, cytoskeleton, proteins, and calcium handling, leading to disturbances in coordinated contraction and subsequent pump failure (55). AAS use and resultant cardiac hypertrophy also derange electrical conduction properties. Prolonged action potential duration is a common feature of myocardial hypertrophy, irrespective of the cause. The resulting electrophysiological disruption results in weaker contractility and increased arrhythmogenicity (56). Therefore, chronic AAS use is associated with hypertrophic and fibrotic cardiac tissue changes, which lead to disturbances in regular coordinated contraction and subsequent pump failure.

##### 1.3.2.2. Role of AAS in right ventricular strain

The impact of AAS use on right ventricle (RV) remains unclear, regardless of its key role in global cardiac function. Kasikcioglu et al. (51) were able to identify RV dysfunction in their study that showed results similar to the effect of AAS on LV function in that the major RV dysfunction that was identified in AAS users was diastolic dysfunction. Alizade et al. (57) showed in their study that despite normal RV systolic function as measured by conventional echo parameters (tricuspid annular plane systolic excursion, RV fractional area change, RV myocardial performance index, RV systolic excursion velocity); two-dimensional speckle tracking echocardiography parameters (RV strain and strain rate) were decreased in AAS users compared to non-users. The combined effect of AAS use and exercise training has been shown to increase the activity of lysosomal hydrolytic enzymes and collagen concentrations in the RV wall (58). Furthermore, Nieminen et al. (58) observed diffuse myocardial fibrosis in two biopsy specimens obtained from the RV of three AAS user weightlifters. These findings indicate that, just as in the case of LV, long-term AAS use also results in fibrosis in the RV, but the responsible molecular mechanisms remain unclear.

##### 1.3.2.3. Role of AAS in in reduction of left ventricular ejection fraction

Most studies that explore the impact of AAS use on LV function have focused on demonstrating LV diastolic dysfunction. However, data on the impact of AAS on LV systolic function and ejection fraction remain limited. Di Bello et al. (59) analyzed regional function in weightlifters using ultrasonic myocardial backscatter to demonstrate that the cyclic variation index of the myocardial echo amplitude of the septum and left ventricular posterior wall was significantly lower for AAS users compared to weightlifters who did not use AAS and normal subjects. The study highlights that despite normal standard echo parameters, Doppler myocardial imaging and strain rate imaging are useful in detecting underlying LV myocardial dysfunction in people using AAS, and that the duration and dose of AAS use were independent determinants of impaired strain rate. The alteration of LV relaxation properties leading to diastolic dysfunction has been attributed to the increased systemic blood pressure observed in AAS users. However, since AAS has also been shown to cause RV diastolic dysfunction irrespective of pulmonary artery pressure, a different explanation for the diastolic dysfunction could be direct AAS-induced structural and/or mechanical alteration of the myocardium.

The role of AAS in reducing left ventricular ejection fraction (LVEF) has gained attention due to the increasing use of advanced cardiac imaging techniques. Baggish et al. (12) found that AAS use can lead to significant impairment in LV systolic dysfunction by both relative (compared to AAS non user's cohort) and absolute standards (as defined by clinical practice). This is a novel finding, as previous studies have not consistently identified a significant impact of AAS use on LVEF. Additionally, the study found no clear relationship between the total amount of AAS used and the severity of cardiac dysfunction, suggesting that the degree of cardiotoxicity from AAS use may be partially and unpredictably related to the lifetime dose.

The impact of AAS use on LV systolic function was further explored by Luijkx et al. (60) using cardiac MRI. The study observed a significantly lower ejection fraction of both ventricles (LV/RV EF 51%/48% vs. 55–57/51%–52%) compared to non-athletes and athletes who did not use AAS. Recently, a study presented at the European Society of Cardiology meeting in 2019 (61) showed that the ejection fraction of AAS users was significantly lower at 49% than at 53%. However, the findings have not been officially published, as they were observed as part of a broader study analyzing the influence of AAS use. Therefore and according to current literature, AAS use can cause depressed LV systolic function and lower LV Ejection Fraction compared to the prior assumption that the effects were largely limited to diastolic dysfunction only.

### 1.4. Diagnosis of AAS induced cardiomyopathy

**Figure 2:**
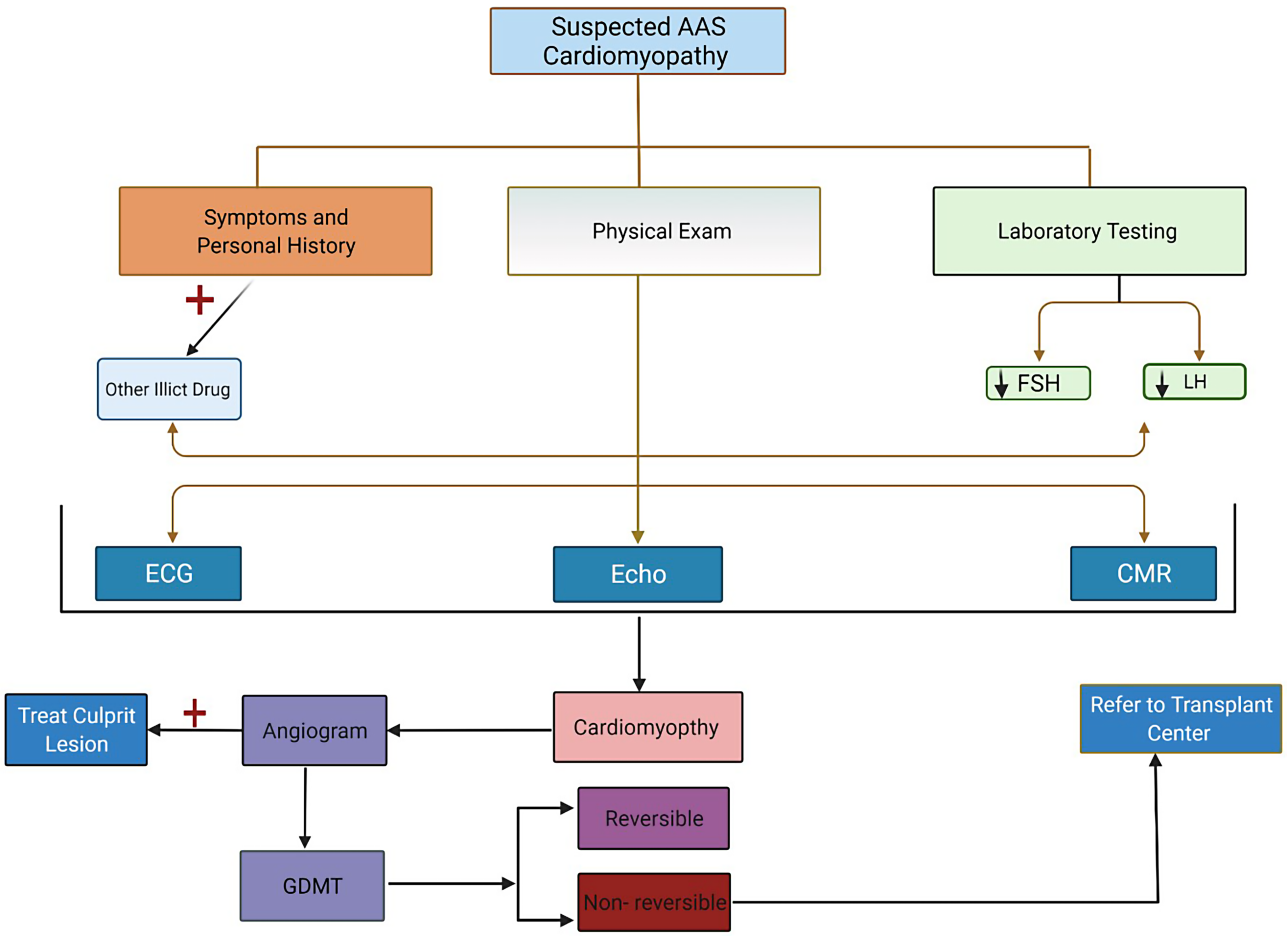
Recommendation for evaluation, testing, and management algorithm for patients suspected of having anabolic androgenic steroids cardiomyopathy.

The diagnosis of AAS-associated cardiomyopathy involves a multistep approach, including a thorough history of AAS use, laboratory analysis of AAS levels where possible, and both imaging and invasive modalities to assess cardiac dysfunction. Figure 2 illustrates diagnostic and treatment algorithm for suspected AAS users. When there is a low threshold for suspected AAS misuse, the history should cover the drug type, dose, mode of consumption, and duration. While a strong therapeutic alliance is the ideal way to obtain information that patients may be reluctant to offer, proposed structured interview modules to assess AAS misuse have also been suggested, showing preliminary reliability and validity (62). Investigations into the use of other substances commonly abused in association with AAS, such as tobacco, alcohol, and recreational drugs, should also be performed when AAS misuse is suspected (26).

The use of AASs can be quantified in several ways. If AAS misuse is suspected but unconfirmed, laboratory analysis may be initiated with serum measurement of substances more readily testable than AAS, including testosterone, follicle-stimulating hormone (FSH), and luteinizing hormone (LH), all of which are decreased in the setting of AAS use (63). While an “athlete biological passport” developed by the World Anti-Doping Agency for elite athletes measures levels of testosterone, testosterone products, and precursors from a baseline level, there is no standard method for measuring AAS in the general population. Various urine and serum assays can be performed to measure specific AAS levels using chromatography-mass spectrometry, enzyme-linked immunosorbent assays (ELISA), and other methods (64, 65). The conversion of known AAS drugs to testosterone equivalents can also be used to estimate AAS exposure over a given period of time (26).

Cardiac dysfunction associated with AAS, including ischemia, left ventricular hypertrophy, reduced EF, and right ventricular strain, is determined in various ways. Transthoracic echocardiography (TTE) is used to evaluate these parameters and assess biventricular size and function (12). Transesophageal echocardiography (TEE) is capable of more extensive measurements such as LV end-diastolic dimension and volume, end-systolic dimension and volume, global longitudinal strain, and myocardial performance index, among other indices (47).

Once cardiomyopathy is diagnosed, coronary computed tomography angiography (CCTA) and cardiac catheterization can help characterize the ischemic vs. non-ischemic nature of the pathology, analyzing coronary artery stenosis, and the degree of atherosclerotic plaque burden (26). Table 3 summarize used modalities in the diagnosis of AAS induced cardiomyopathy.

**Table 3:** Diagnostic modalities.

Tool Study	Findings	Comment	Future need
Laboratory Testing	Decreased levels of testosterone, (FSH), and (LH) Elevated AAS	Currently, there are no standardized laboratory tests for AAS detection outside of the World Anti-Doping Agency for professional athletes.	As AAS use became rampant in the public, more widely available testing may be needed
Electrocardiography	Possible left or right ventricular hypertrophy	Can be used to rule out ischemic cardiomyopathy if previous ischemic changes are detected	
Echocardiography	Left and right ventricular hypertrophy, left ventricular systolic and diastolic dysfunction, right ventricular strain	Most commonly use tool for determining cardiac dysfunction in the setting of suspected AAS cardiomyopathy.	Can be used to study reversibility
Cardiac magnetic resonance imaging (CMR)	Reduced left and right ventricular ejection fraction	Advanced imaging tool to further detect cardiac dysfunction	
Angiography	Normal coronary arteries	Can be used to rule out ischemic cardiomyopathy	

### 1.5. Treatment of AAS induced cardiomyopathy

Although ischemic cardiomyopathy is more common than non-ischemic cardiomyopathy, which includes AAS-induced cardiomyopathy, guideline-directed medical therapy (GDMT) for both etiologies is fairly similar (66). First-line GDMT agents fall into four main classes: beta-adrenergic receptor blockers (beta-blockers), renin-angiotensin system inhibitors, including angiotensin receptor neprilysin inhibitors (ARNi), angiotensin-converting enzyme inhibitors (ACEi), angiotensin II receptor blockers (ARBs), mineralocorticoid receptor antagonists (MRAs), and sodium-glucose cotransporter-2 inhibitors (SGLT2i).

All patients with LV dysfunction, regardless of etiology, should receive beta-blockers and ACE-I/ARBs, except those with contraindications or intolerance due to the mortality-reducing benefit provided by both agents (67). Beta-blockers have been used for decades in the management of LV dysfunction because of their ability to inhibit sympathetic nervous system stimulation of myocardial tissue (68, 69). ACE-I/ARBs cause peripheral vasodilation, leading to decreased afterload on the heart and prevention of cardiac remodeling. In particular, ARBs have been shown to decrease the harmful effects of angiotensin II on the cardiovascular system, including vasoconstriction and myocardial fibrosis (67, 70). While previous guidelines recommend ACE-I/ARBs as the mainstay of therapy, the latest HF guidelines include ARNis as the first-line therapy equivalent to ACE-I/ARBs (71). ARNis inhibit neprilysin, an enzyme involved in the degradation of natriuretic peptides and bradykinin, among other peptides. The PARADIGM-HF study compared the ARNi sacubitril/valsartan to enalapril, which showed a 20% reduction in mortality and hospitalizations due to HF with the use of the ARNi compared to the ACE-I (72, 73). MRAs act as antagonists of aldosterone receptors, including spironolactone and eplerenone, both of which confer benefits to morbidity and mortality in HF and are highly economical for patients (72). Additionally, Dapagliflozin in Patients with Heart Failure and Reduced Ejection Fraction (DAPA-HF) (74) and Empagliflozin Outcome Trial in Patients With Chronic Heart Failure and a Reduced Ejection Fraction (EMPEROR-Reduced) trial (75) demonstrated a decrease in hospitalization and all-cause mortality in patients with HF who used SGLT2i. Therefore, the latest guidelines recommend SGLT2is for all patients with symptomatic chronic HF, irrespective of the presence of diabetes (72). Vasodilator combination drugs, such as hydralazine and isosorbide dinitrate, have been shown to improve survival in patients with HF compared to placebo, particularly in self-identified African-American patients who remain refractory to optimal therapy (72, 76). Additional therapies are indicated for patients who present with prior or current symptoms of clinical HF (77, 78).

### 1.6. Is AAS induced cardiomyopathy reversible?

The reversibility of AAS toxicity varies and can depend on user factors, the type, strength, and duration of AAS used, and the organ system involved (21). The effects of AAS on the hypothalamic pituitary adrenal (HPA) axis, for example, have shown reversibility in terms of recovery from oligospermia in many but not all patients (67). In terms of cardiac sequelae of AAS, various studies have shown opposing findings, with some demonstrating reversibility, while others do not. The HAARLEM study followed men aged ≥18 years who intended to use AAS and performed 3D echocardiography before, during, and one year after complete discontinuation of use, demonstrating reversibility in multiple measured parameters. Following a median recovery period of approximately 8 months, the measures of LV function, including LV mass, intraventricular end-diastolic septal thickness, and left ventricular end-diastolic posterior wall thickness, returned to baseline. Additionally, the left atrial volume and ratio of peak velocity of early to late diastolic waves, which had also been aberrant during the period of AAS use, also returned to within the normal range (43). Multiple individual cases have reported improvement in reduced EF as a consequence of AAS use following treatment with GDMT and non-pharmacologic strategies together with medication, such as the placement of left ventricular assist devices (LVAD) (68).

Conversely, other studies have reported contradictory findings indicating the irreversibility of AAS-induced cardiomyopathy. One study of athletes, comparing active users of AAS, former users who had been steroid-free for at least one year, and lifetime non-users, found that even after years of being steroid-free, former users still had slightly concentric left ventricular hypertrophy compared to lifetime non-users (65). However, other studies have reported mixed results regarding reversibility. One retrospective study that reviewed a group of patients with AAS-induced HF over the course of 4 years found that some patients had recovered EF with GDMT within the first six months of diagnosis, whereas others required referrals to cardiac transplantation and had LVAD placement with no improvement in EF (70). Further investigations are needed to evaluate whether reversibility is possible and the underlying mechanism.

## 2. Encouraging general awareness and understanding of inappropriate AAS

Contrary to what the general public and media communities portray (79), AAS use is not primarily limited to competitive athletes. Muscle dysmorphia, also known as “megarexia,” is a dominant risk factor for AAS use (80). One of the major challenges related to this form of drug abuse is that few AAS users seek medical assistance, and certain users do not trust clinicians (81). Furthermore, even when patients present to medical attention with vague symptoms that may arise from AAS use, many practitioners fail to specifically ask about their use, often because they are less acquainted with AAS abuse than with other substances (23, 81).

## 3. Implications of social media and internet commerce on AAS use: urgent need for action

While any form of substance abuse is related to multifactorial causes (82), the media and Internet may play a specific role in fueling the ever-rising incidence of AAS abuse. Muscle dysmorphia is a subset of body dysmorphia (83), and is a preoccupation with a personal muscular appearance that particularly affects men. Up to 9%–25% of all men with body dysmorphia, that is, approximately 2.2% of all men in the US, may be affected by muscle dysmorphia (84). Frequent promotion of weight control and muscle development in multimedia has been linked to eating and behavioral disorders, including body dysmorphia (85–87). There is evidence that, not only brief exposure to media images of muscularity leads to men finding dissatisfaction with their own physiques (88), but also the internalization of media portrayals of muscular men may be the leading predictive factor in changes to behavior and methods of achieving such unrealistic body types, including AAS misuse (84, 89–91).

Arguably, one of the biggest challenges in addressing contemporary AAS abuse in modern times is the rise of Internet-based commerce, which is the most frequently used medium for obtaining AAS (92). Consumers are now capable of placing their orders for AAS online, and deliveries can be made from countries where a prescription is not required or from sources that circumvent existing regulations. One study demonstrated that when searching for typical terms related to AAS on the Internet, less than 5% of such websites provide information regarding the safety of AAS, abuse potential, or resources for cessation (92). Therefore, it is crucial for clinicians to be especially aware of the methods and specific types of AAS that patients may be able to obtain online and to continue educating their patients against such false purchases over the Internet.

## 4. Conclusions

In summary, HF has a significant impact on both health and the economy, and as such, has gained attention in various fields, including healthcare policy and public health. AAS-induced cardiomyopathy is a potentially under-investigated cause of non-ischemic cardiomyopathy and HF due to the lack of familiarity with this issue and many other common causes of the disease. It is essential to raise awareness regarding the prevalence of AAS use, its various manifestations, and the potential consequences of its toxicity, as well as to develop clear guidelines for its management. Addressing the underlying cause of HF is crucial for managing the disease and reducing its exacerbations. Additional research on the specific mechanisms underlying AAS-induced cardiomyopathy and risk stratification in the presence of the disease will provide a foundation for targeted and effective treatments. There is some evidence that AAS-induced cardiomyopathy can be reversible; however, more research is needed to determine the degree of reversibility and whether current HF management needs to be adjusted to address this population, specifically through long-term prospective studies.
